# Lessons learned: challenges in recruiting and engaging people with heavy cannabis use for online interventions in Canada

**DOI:** 10.1186/s42238-026-00425-9

**Published:** 2026-04-16

**Authors:** Karli K. Rysen, Corey S. Mackenzie, Julian M. Carusone, Michael P. Schaub, Andreas Wenger, Harold Wallbridge, Jason D. Edgerton, Richard Kruk, Matthew T. Keough

**Affiliations:** 1 Department of Psychology, University of Manitoba, 190 Dysart Rd., Winnipeg, MB R3T 2N2 Canada; 2 Department of Psychology, York University, Toronto, ON Canada; 3 Swiss Research Institute for Public Health and Addiction, University of Zurich, Zurich, Switzerland; 4 Department of Sociology and Criminology, University of Manitoba, Winnipeg, MB Canada; 5 Senior Scientist, Homewood Research Institute, 150 Delhi St, Guelph, ON Canada; 6 Department of Clinical Health Psychology, Max Rady College of Medicine, University of Manitoba, Winnipeg, MB Canada

**Keywords:** Cannabis, Online intervention, Recruitment, Treatment initiation, Treatment engagement

## Abstract

**Background:**

People who seek treatment often disengage between completing screening and starting treatment. Among those who begin treatment, many have low completion of program content and are lost to follow up. Currently, little is known about predictive factors of treatment initiation and engagement. The aims of this secondary analysis of the Canadian *CANreduce* program were to discuss the practicality of a randomized controlled trial for online heavy cannabis use treatment and to examine predictors of treatment initiation and engagement in the Canadian *CANreduce* program.

**Method:**

The *CANreduce* intervention was pre-registered on clinicaltrials.gov for traceability (ID: NCT04965012). Statistical models were organized into three conceptual predictor groupings using baseline data: individual cannabis-specific factors, mental health and other substance use factors, and treatment belief factors. Binary logistic regressions examined which factors predicted treatment initiation in the *CANreduce* treatment program and multiple regression analyses examined which factors predicted percentage of the *CANreduce* program modules completed among participants who initiated treatment.

**Results:**

Despite following the core elements of published treatment retention protocols, challenges in recruitment were evident. Of 928 people who created a profile on the website and began screeners, 86.3% (*n* = 801) completed screeners. Of the 801 who completed screeners, 31.3% (*n* = 251) were eligible for the program. Of those eligible and assigned to active treatment, 54.3% (*n* = 51) assigned to the therapist condition and 45.7% (*n* = 43) assigned to the research assistant condition initiated treatment. Treatment initiation predictors included higher cannabis use problems score, lower family history density, increased alcohol use frequency, and more positive attitudes towards treatment. Treatment engagement (percentage of program completed) predictors included increased social motives for cannabis use and more positive attitudes towards treatment.

**Discussion:**

This secondary analysis shifts the focus from treatment efficacy to pre-treatment attrition and early engagement in an online cannabis intervention. Substantial recruitment and initiation challenges were observed, highlighting vulnerabilities in the treatment-seeking pathway before content is accessed. By identifying predictors of treatment initiation and module completion, this study offers practical “lessons learned” to inform recruitment and engagement strategies in future online cannabis trials.

## Introduction

Over time, the demand for cannabis use treatment has increased (Manthey et al. 2021). Data compiled by the World Health Organization (WHO) indicates cannabis is second only to alcohol for a reason for substance use treatment entry (World Health Organization 2016). In Western countries, it is estimated that one quarter of individuals entering addiction treatment report cannabis problems, and these individuals are typically young men with a high rate of legal problems (Rush & Urbanoski 2007). Literature specifically on Canadian treatment seeking rates and individual factors is sparse and often only examines cannabis use being reported as a comorbidity of other substance use (Williamson et al. 2022). Despite the overall increase in need for cannabis use treatment programs, little information is known about the individual factors affecting treatment seeking and treatment engagement.

First, many individuals who experience Cannabis Use Disorder (CUD) do not seek treatment (Roffman & Stephens 2006), and even those who do may drop out prematurely. Premature treatment dropout is a problem that exists across all substance use treatments, not just for cannabis, with studies citing attrition rates for substance use treatments ranging from 30 to 66% (Etter 2005; Frohlich et al. 2022; Lappan et al. 2020; Schaub et al. 2015). Factors that may interfere with accessing and completing evidence-based treatment when it is available include worries about stigma (Gates et al., 2016; Monaghan et al. 2016; van der Pol et al. 2013), cost of treatment (Ellingstad et al. 2006), and complicated access in rural or remote areas (Richards & Viganó 2013). A large body of research examining best practices for maximizing participant retention and increasing engagement in substance use treatment once it begins cites as participant retention as a common issue (Eysenbach 2005; Etter 2005; Frohlich et al. 2022; Rysen et al., under review). However, many individuals drop out of cannabis use treatment in the period between completing screening and initiating the program. Cannabis use studies tend to show significant reduction from those who are interested and eligible for treatment, and those who complete the treatment (Norberg et al. 2012). Tossman and colleagues (2011) noted that out of the 863 participants recruited for their cannabis use treatment program and allocated to the intervention condition, 58% did not end up taking part of the study. Non-initiation of treatment can be further complicated by being placed on a waitlist, a key aspect of gold-standard RCTs, as literature demonstrates that shorter wait times between assessment and treatment increase odds of participating in treatment (Claus & Kindleberger, 2002). Despite the common difficulty of people dropping out of programs between screening and treatment, little literature exists examining the factors that contribute to pre-treatment drop out specifically in cannabis treatment programs. A 2002 study by Vendetti and colleagues examined pre-treatment drop out factors among 813 eligible study participants with cannabis dependence, finding pre-treatment drop out was associated with being younger, unmarried, unemployed, less educated, and being of Asian American or Native American descent. These authors also found that having greater self-perceived dependence on marijuana and using other drugs were associated with not initiating cannabis treatment (Vendetti et al. 2002). Given the widely prevalent problem of treatment non-initiation, more research is needed examining factors that predict pre-treatment disengagement.

Once an individual begins a treatment program, low engagement with the program is also a commonly cited issue in substance use research. Discontinuation of treatment is common, with some researchers citing approximately 20–70% discontinuation of general psychosocial treatment (Gearing et al. 2014), and 50% discontinuation over the course of cannabis use treatment (Tossman et al., 2011). Similarly, Sinadinovic and colleagues (2020) found that 35% of their cannabis intervention group participants did not visit the treatment website after the first day. In terms of treatment content completion, the extant cannabis treatment literature reports a range of module completions, including 3.9/13 modules (30%; Sinadinovic et al. 2020), 3.2/8 modules (40%; Schaub et al. 2015), 4.1/8 (51.58%, Rysen et al. [2026], under review) and 3.5/6 modules (58.33% Rooke et al. 2013). Given that adherence to treatment programs has been demonstrated to increase positive outcomes generally in mental health treatment (Gearing et al. 2014), and lower cannabis use disorder symptoms at follow up in cannabis use treatments (Sinadinovic et al. 2020), examining factors that predict engagement in treatment is a much-needed area of research.

Drawing on wider literature, several factors have been demonstrated to impact general engagement and outcomes in mental health and substance use treatments. Sociodemographic factors, specifically gender and ethnicity, are frequently reported predictors of treatment outcomes (Gergov et al. 2024). While gender findings are inconsistent for which gender benefits most from treatment, studies suggest ethnic minority status may predict poorer treatment outcomes (Gergov et al. 2024). Beyond sociodemographic factors, several key factors may impact treatment initiation and engagement, including severity of substance use disorder (Brown et al., 2011; Sinadinovic et al. 2020); motives for using substances (Dow & Kelly 2013); using other substances (Subbaraman et al. 2017); other mental health difficulties (Compton et al. 2003); family history density (Khoddam et al. 2015); motivation for treatment (Alfonsson et al. 2016); and attitudes towards treatment (Pettinati et al. 2003).

To date, there have been limited studies examining the predictors of treatment initiation and engagement for online cannabis use treatments. While studies examining the predictors of treatment initiation and engagement exist in other similar areas of research (i.e., Brown and colleagues (2011) examining predictors of treatment initiation and engagement in co-occurring serious mental illness and substance use disorders), there is a relative scarcity of cannabis-specific research. Hence, the goals of the present paper were to 1) discuss the practicality of a randomized controlled trial for online heavy cannabis use treatment and to 2) to examine predictors of treatment initiation and engagement in the Canadian *CANreduce* program.

Based on extant literature, the hypotheses were as follows:

1. Hypothesis 1: Regarding individual cannabis-specific factors, higher treatment initiation and program engagement would be predicted by increased scores on the CUDIT, lower family history density, and higher cannabis motives.
2. Hypothesis 2: Regarding mental health and other substance use factors, higher treatment initiation and program engagement would be predicted by increased depression, presence of a diagnosed mental illness, presence of lifetime mental illness treatment, increased alcohol use frequency, and increased alcohol use quantity
3. Hypothesis 3: Regarding treatment belief factors, higher treatment initiation and engagement would be predicted by more positive attitudes towards treatment seeking, and higher pre-treatment importance, confidence and readiness.

## Methods

### *CANreduce* study overview

Full *CANreduce* methodology can be found in a paper entitled, “*Evidence-Based Therapist Guided Introduction to Online Heavy Cannabis Use Treatment in Canadian Adults: A Randomized Controlled Trial (RCT)*” (Rysen et al., under review), which examines the primary study outcomes of the trial. Only relevant details to the secondary analysis regarding treatment initiation and drop out will be reiterated here. The self-guided *CANreduce* program is comprised of eight modules containing strategies of cognitive behavioural therapy and motivational interviewing approaches to help participants think about reasons for changing their cannabis use habits; consider benefits and harms of their current level of use, identify goals for cannabis use reduction; learn coping strategies for cravings, triggers, and social pressures; and learn how to prevent slips. Eligible participants were randomized into one of three conditions: (1) a one-hour Motivational Enhancement Therapy (MET)-therapist guided introduction plus 6-week, online, self-guided treatment program; (2) a 15-min non-MET research assistant introduction plus 6-week, online, self-guided treatment program; or (3) a psychoeducational control condition.

### Preintervention assessment battery

All participants registering for an account were given a comprehensive questionnaire battery, serving as a baseline assessment for the present treatment study. Additional measures not included in the present study were collected as part of a larger data set. Three conceptual predictor groupings using information collected during baseline screening (i.e., to determine eligibility, prior to randomization) were organized based on extant literature to examine predictors of treatment initiation and engagement in the Canadian *CANreduce* program as outlined below.

### Individual cannabis-specific factors

#### Cannabis use disorder severity

The Cannabis Use Disorders Identification Test-Revised (CUDIT-R; Adamson et al. 2010) was used to assess cannabis use disorder severity at baseline. The CUDIT-R is an 8-item self-report measure. Participants were asked to rate frequency of cannabis-related problems or concerns on a scale from 0 to 4. Sum scores were calculated, where higher scores indicated more problematic cannabis use. The internal consistency of the CUDIT-R at baseline was acceptable (α = 0.75).

#### Family history density

Family history density was calculated from information collected in the demographic questionnaire, which asked “Do you think your biological mother/biological father/biological grandparents have/had cannabis use problems?” A weighted score was created, similar to alcohol use family history density research by Stoltenberg and colleagues (1998), where both parents and grandparents (i.e., each alcoholic parent received a score of 0.5, and each alcoholic grandparent received a score of 0.25, for a range of 0–2) were considered. However, since our question simply asked if “biological grandparents” as a collective unit had cannabis problems, and not each specific grandparent in a separate question, our calculation and sum score was slightly different than Stoltenberg and colleagues (1998). Parents with probable cannabis use problems were assigned a score of 0.5 each, and endorsement of any grandparent with a probable cannabis use problem was assigned a score of 0.25, for a total possible range of 0 (indicating no family history density) to 1.25 (high family history density).

#### Cannabis motives

The Marijuana Motives Questionnaire (MMQ; Simons et al. 1998) is a 25-item measures of motives for marijuana use. Participants were asked to indicate the frequency with which they use marijuana for various reasons on a scale from 1 (Almost never or never) to 5 (Almost always or always). Subscales were calculated for the 5 factors: enhancement (e.g., *“Because it gives me a pleasant feeling”*), conformity (e.g., *“To fit in with the group I like”*), expansion (e.g., *“To be more open to experiences”*), coping (e.g., *“To forget about my problems”*) and social motives (e.g., *“Because it makes social gatherings more fun*”), where higher scores indicate higher rated motives in each subscale. The internal consistency of the MMQ subscales were all good (enhancement, α = 0.81; conformity, α = 0.83; coping, α = 0.83; social, α = 0.82) or excellent (expansion, α = 0.93).

### Mental health and other substance use factors

#### Depression

Depression was assessed using the Patient Health Questionnaire (PHQ-9; Kroenke et al. 2001). Participants were asked to rate how much they had been bothered by depressive symptoms on a scale of 0 (not at all) to 3 (nearly every day). Sum scores were calculated where increased score indicated higher depressive symptoms. The PHQ-9 internal consistency was good (α = 0.89).

#### Diagnosed mental illness

Participants self-reported the presence of diagnosed mental illness in the demographics questionnaire (0 = no, 1 = yes). Participants were also given the opportunity to specify mental illness diagnosis, but this information was not included in the present analyses.

#### Lifetime mental illness treatment

Participants self-reported the presence of lifetime treatment for mental illness in the demographics questionnaire (0 = no, 1 = yes). Participants were also given the opportunity to specify mental illness treatment type, but this information was not included in the present analyses.

#### Alcohol use frequency

Alcohol use frequency was assessed using one question on the National Institute on Alcohol Abuse and Alcoholism’s (NIAAA 2007) recommended alcohol use disorder screening questions. Participants were asked how frequently they consumed any kind of drink containing alcohol, with options ranging from never (0) to every day (7). Higher scores indicated higher alcohol use frequency.

#### Alcohol use quantity

Alcohol use quantity was assessed using one question on the National Institute on Alcohol Abuse and Alcoholism’s (NIAAA 2007) recommended alcohol use disorder screening questions. Participants were asked how many standard alcoholic drinks they had on a typical day they drank alcohol, with options ranging from 1 (1) to 25 or more (10). Higher scores indicated higher alcohol use quantity.

### Treatment belief factors

#### Attitudes towards treatment seeking

The Attitudes Towards Seeking Professional Psychological Help – Short Form (ATSPPH-SF; Fischer & Farina 1995) is a 10-item self-report measure that was used to assess attitudes towards seeking treatment. Participants were asked to indicate how strongly each statement about treatment seeking beliefs applies to them on a scale from 1 (Strongly Disagree) to 5 (Strongly Agree). Sum scores were calculated, where higher scores indicated more positive attitudes towards seeking professional psychological help. The ATSPPH-SF internal consistency was good (α = 0.85).

#### Pre-treatment importance, confidence and readiness

Scores of the participants’ pre-treatment self-reported importance, confidence and readiness (DiClemente et al. 2004) were collected by asking participants how important treatment was, how confident they were in their ability to change, and how ready they were to change at that point in time. Possible scores ranged from 0 (Not confident/important/ready) to 10 (Very important/confident/ready). Scores were reported separately for importance, confidence and readiness, no sum scores were created.

### Data analytic plan

Data were analyzed using SPSS version 25.0. Statistical models were organized into three conceptual predictor groupings based on the literature: individual cannabis-specific factors, mental health and other substance use factors, and treatment belief factors. We opted for three separate conceptual models for each outcome because we had a modest sample size, and thus, lacked power to examine one larger combined model. Individual cannabis-specific factors included cannabis problems as measured by the CUDIT-R, family history density, and cannabis motives including for enhancement, conformity, expansion, coping, and social factors as measured by the MMQ. Mental health and other substance use factors included depression as measured by the PHQ-9, diagnosed mental illness, lifetime mental illness treatment, alcohol use frequency and alcohol use quantity as measured by the NIAAA. Treatment belief factors included attitudes towards treatment as measured by the ATSPPH-SF, pre-treatment importance, pre-treatment confidence and pre-treatment readiness. Given that gender (Callaghan et al. 2019; Dacosta-Sánchez, et al., 2023; Fairman et al. 2019; Feingold et al. 2024; Rotermann 2020) and race (Fairman et al., 2014; Feingold et al. 2024; Peters et al. 2014) have been demonstrated to be significant factors in the onset, severity and treatment success of heavy cannabis use, both were included as covariates throughout each of the models explained below. Dichotomous covariates were organized in terms of gender (0 = men, 1 = women^1^) and race (0 = white, 1 = non-white). We did not have missing data on the baseline variables analyzed in this secondary analysis.

First, binary logistic regressions examined which factors predicted treatment initiation in the *CANreduce* treatment program. Group one was defined as those who were eligible for the program but did not attend the introductory session or have access to the program. Group two was defined as those who were eligible and participated in either the therapist-guided introduction or research assistant-guided introduction and received subsequent access to the program. Model fit information was examined to explore if the fit of the model was improved with the addition of predictor variables, and then individual variable contributions were examined.

Second, multiple regression analyses examined which factors predicted engagement in the program, as defined by greater percentage of the *CANreduce* program modules completed. Percentage of program completed was calculated examining how many cumulative pages of modules were completed. Only participants who engaged in the program (i.e., attended either the therapist-guided introduction or the research assistant-guided introduction) gained access to the program, and hence, only these participants were included in this portion of the analysis. We examined the proportion of variance explained in the outcome by the predictors as a set and then individual variable contributions were examined. Variables were entered in a hierarchical method, with two blocks of variables. The first block included covariates of gender and race as predictors, with percentage of program completion as the dependent variable. In block two for each conceptual predictor grouping, the predictors of interest were examined.

## Results

### Recruitment

**Fig. 1:**
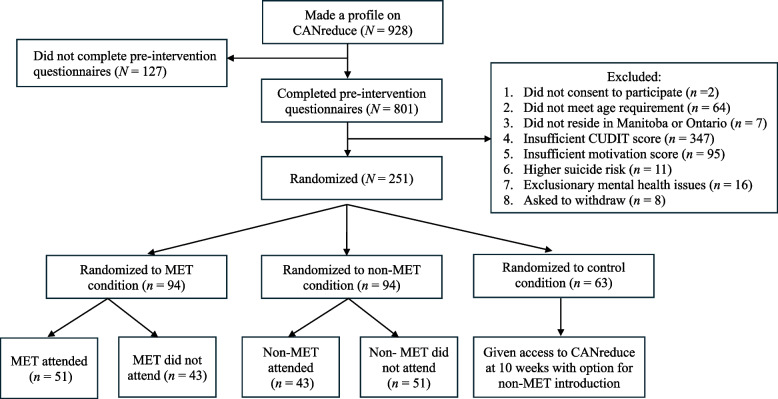
Trial flow chart

Refer to Fig. 1 for comprehensive flow chart. Over nine months of active recruitment resulted in 928 individuals interested in the *CANreduce* treatment program creating a profile on the *CANreduce* website and beginning to fill out the pre-intervention assessment battery. Out of 928, 801 (86.3%) completed the battery, indicating that 127 (13.7%) individuals (who were potentially eligible for the program) did not complete the pre-intervention battery. Of the 801 who completed questionnaires, 550 (68.7%) were excluded for the following reasons: did not consent to participate (*n* = 2), did not meet age requirement (*n* = 64), did not reside in Manitoba or Ontario (*n* = 7), insufficient CUDIT-R score (*n* = 347), insufficient motivation score (*n* = 95), higher suicide risk (*n* = 11), exclusionary mental health issues (*n* = 16) and voluntary withdrawal from study (*n* = 8). Removing ineligible participants from the sample left 251 (31.3%) eligible participants that were randomly assigned via random number generator into one of three conditions: MET-therapist guided introduction and access to the treatment program (*n* = 94); non-MET research assistant guided introduction and access to the treatment program (*n* = 94) and waitlist control (*n* = 63). Of participants randomly assigned to the MET-therapist guided introduction, 51 (54.3%) attended the introduction to the program with the MET-therapist, and 43 (45.7%) did not attend (which included either no response to facilitator email or no-showing their arranged appointment). Of participants randomly assigned to the non-MET research assistant guided introduction, 43 (45.7%) attended the introduction to the program with the non-MET research assistant, and 51 (54.3%) did not attend (which included either no response to facilitator email or no-showing their arranged appointment). This indicates that 94 participants (50%) attended either an MET-therapist or non-MET research assistant introduction, and an additional 94 participants (50%) were eligible but did not attend an introduction. All participants randomly assigned to the waitlist control received automatic access to the *CANreduce* treatment program at 10 weeks regardless of questionnaire completion at end of waiting period or follow up, or further interaction with facilitators. All participants in the waitlist control (*n* = 63) were also offered a non-MET research assistant introductory meeting after the allotted 10-week waiting period, but only 4 people (6.3%) completed this meeting. Overall, only 10.1% of participants (*n* = 94) from the full recruitment sample (*N* = 928), or 50% of eligible participants across randomized treatment groups (*n* = 94), engaged in one of the two *CANreduce* guided introductions and subsequent treatment program (Table 1).

**Table 1:** Descriptive statistics of study variables by group at baseline

Variable	Grouping	
	Eligible who attended (*n* = 94)	Eligible with no participation (*n* = 94)
Age, *M* (SD)	32.05 (10.92)	27.44 (9.08)
Gender, % (*n*)		
Man	38.3% (36)	34% (32)
Woman	58.5% (55)	63.8% (60)
Transgender	2.1% (2)	1.1% (1)
Non-binary	1.1% (1)	1.1% (1)
Race, % (*n*)		
East Asian, South-East Asian, Pacific Islander (e.g., Chinese, Japanese, Korean, Vietnamese, Thai)	5.3% (5)	7.4% (7)
Middle Eastern, North African, Central Asian (e.g., Jordanian, Saudi, Egyptian, Moroccan, Iranian)	6.4% (6)	5.3% (5)
Hispanic or Latino (e.g., Brazilian, Chilean, Mexican, Cuban)	1.1% (1)	3.2% (3)
Caucasian or White (e.g., Russian, German, Latvian, French, Scottish, Italian)	70.2% (66)	53.2% (50)
Black (e.g., African- American, Nigerian, Haitian, Jamaican, Somali)	4.3% (4)	10.6% (10)
Indigenous or Aboriginal (e.g., First Nations, Inuit, Metis, Native American, Native Australian)	7.4% (7)	6.4% (6)
South Asian (e.g., Indian, Pakistani, Sri Lankan, Nepalese)	4.3% (4)	9.6% (9)
Other	1.1% (1)	4.3% (4)
Pre-treatment Importance, *M* (SD)	9.09 (1.27)	8.88 (1.34)
Pre-treatment Confidence, *M* (SD)	5.85 (2.31)	6.51 (2.61)
Pre-treatment Readiness, *M* (SD)	7.69 (2.13)	7.97 (2.10)
NIAAA Alcohol frequency, *M* (SD)	2.69 (2.19)	2.09 (1.88)
NIAAA Alcohol quantity, *M* (SD)	2.69 (1.28)	2.91 (1.31)
CUDIT-R, *M* (SD)	22.54 (5.57)	20.38 (6.35)
PHQ, *M* (SD)	13.72 (6.67)	14.30 (6.92)
MMQ Enhancement motives, *M* (SD)	2.92 (0.69)	2.75 (0.84)
MMQ Conformity motives, *M* (SD)	1.13 (0.26)	1.24 (0.54)
MMQ Expansion motives, *M* (SD)	1.99 (0.94)	2.07 (1.03)
MMQ Coping motives, *M* (SD)	2.96 (0.79)	2.96 (0.90)
MMQ Social motives, *M* (SD)	1.93 (0.79)	1.96 (0.82)
ATSPPH-SF, *M* (SD)	40. 31 (6.30)	36.72 (7.03)
Family history density, *M* (SD)	0.14 (0.27)	0.19 (0.32)
Mental Illness Diagnosis, % (*n*)		
No	35.1% (33)	48.9% (46)
Yes	63.8% (60)	51.1% (48)
Mental illness treatment history, % (*n*)		
No	37.2% (35)	51.6% (48)
Yes	61.7% (58)	47.9% (45)

*PHQ-9* Patient Health Questionnaire – 9, *NIAAA* National Institute on Alcohol Abuse and Alcoholism’s recommended alcohol use disorder screening questions, *CUDIT-R* Cannabis Use Disorder Identification Test – Revised, *MMQ* Marijuana Motives Questionnaire, *ATSPPH-SF* Attitudes Toward Seeking Professional Psychological Help- Short Form

### Predictors of initiating treatment

Binary logistic regression was performed to see if conceptual predictor groupings predicted the odds of an individual initiating participation in the *CANreduce* treatment program, after controlling for gender and race. The reference group was those who attended the introductory session (coded as 0) compared to those who were eligible but did not attend (coded as 1), hence negative coefficients indicated increased likelihood of attending. Relative to the intercept-only model, the inclusion of individual cannabis-specific factors significantly improved model fit (*X*^2^ [9, *N* = 188] = 22.74, *p* = 0.007). Collectively, the individual cannabis-specific factors explained an estimated 16% (Nagelkerke pseudo *R*^2^ = 0.162) of the variance in likelihood of attending the program. Results showed that having baseline higher cannabis use problems and lower^2^ family density history increased the odds of participating in the *CANreduce* program (see Table 2). Relative to the intercept-only model, the inclusion of mental health and other substance use factors did not significantly improve model fit (*X*^2^ [7, *N* = 188] = 13.20, *p* = 0.067). Collectively, the inclusion of mental health and other substance use factors explained 13% (Nagelkerke pseudo *R*^2^ = 0.131) of the variance in likelihood of attending the program. After controlling for race and gender, results showed increased alcohol use frequency increased the odds of participating in the *CANreduce* program (see Table 3). Relative to the intercept model, the inclusion of treatment belief factors significantly improved model fit (*X*^2^ [6, *N* = 188] = 18.07, *p* = 0.006). Collectively, the treatment belief factors explained 13% (Nagelkerke pseudo *R*^2^ = 0.132) of the variance in likelihood of attending the program. Results showed more positive attitudes towards seeking psychological help increased the odds of participating in the *CANreduce* program (see Table 4).

**Table 2:** Binary logistic regression results for individual cannabis factors predicting treatment initiation

Factor	*B*	Std. Error	Wald x^2^	df	Sig	Odds Ratio [95% CI]
Intercept	1.623	1.117	2.112	1	.146	
Gender (Cov)	−0.214	0.351	0.373	1	.541	0.807 [0.406—1.605]
Race (Cov)	−0.479	0.341	1.972	1	.160	0.619 [0.317—1.209]
Cannabis problems (CUDIT)	**−0.093**	**0.033**	**7.969**	**1**	.005**	**0.911 [0.854—0.972]**
Family history density	**1.300**	**0.607**	**4.580**	**1**	.032*	**3.669 [1.116—12.066]**
MMQ Enhancement motives	−0.555	0.288	3.727	1	.054	0.574 [0.327—1.008]
MMQ Conformity motives	0.702	0.477	2.167	1	.141	2.017 [0.792—5.133]
MMQ Expansion motives	0.095	0.215	0.195	1	.658	1.100 [0.722—1.676]
MMQ Coping motives	0.372	0.229	2.637	1	.104	1.451 [0.926—2.275]
MMQ Social motives	0.152	0.263	0.333	1	.564	1.164 [0.695—1.950]

*Cov* Covariate, *CUDIT-R* Cannabis Use Disorder Identification Test – Revised, *MMQ* Marijuana Motives Questionnaire

Gender coded as 0 = men, 1 = women; and race coded as 0 = white, 1 = non-white

Substantive significant predictors are bolded where *denotes significance at 0.05 level; **denotes significance at 0.01 level. The Nagelkerke pseudo *R*^2^ for this model was 0.162

**Table 3:** Binary logistic regression results for mental health and other substance use factors predicting treatment initiation

Factor	*B*	Std. Error	Wald x^2^	df	Sig	Odds Ratio [95% CI]
Intercept	0.265	0.905	0.086	1	.770	
Gender (Cov)	0.298	0.397	0.564	1	.453	1.347 [0.619—2.932]
Race (Cov)	−0.810	0.414	3.834	1	.050*	0.445 [0.198—1.001]
Alcohol use frequency (NIAAA)	**−0.249**	**0.123**	**4.072**	**1**	.044*	**0.779 [0.612—0.993]**
Alcohol use quantity (NIAAA)	0.276	0.158	3.038	1	.081	1.317 [0.996—1.796]
Diagnosed mental illness	−0.708	0.573	1.528	1	.216	0.493 [0.160—1.514]
Lifetime mental illness treatment	0.404	0.592	0.466	1	.495	1.498 [0.469—4.784]
Depression (PHQ-9)	−0.005	0.030	0.031	1	.861	0.995 [0.938—1.055]

*Cov* Covariate, *PHQ-9* Patient Health Questionnaire – 9, *NIAAA* National Institute on Alcohol Abuse and Alcoholism’s recommended alcohol use disorder screening questions

Gender coded as 0 = men, 1 = women; and race coded as 0 = white, 1 = non-white

Substantive significant predictors are bolded where *denotes significance at 0.05 level; **denotes significance at 0.01 level. The Nagelkerke pseudo *R*^2^ for this model was 0.131

**Table 4:** Binary logistic regression results for treatment belief factors predicting treatment initiation

Variables	*B*	Std. Error	Wald x^2^	df	Sig	Odds Ratio [95% CI]
Intercept	2.663	1.521	3.064	1	.080	
Gender (Cov)	0.357	0.338	1.116	1	.291	1.429 [0.737—2.772]
Race (Cov)	−0.442	0.342	1.670	1	.196	0.643 [0.329—1.257]
Attitudes towards treatment (ATSPPH-SF)	**−0.078**	**0.027**	**8.453**	**1**	.004**	**0.925 [0.877—0.975]**
Pre-treatment importance	−0.088	0.131	0.456	1	.500	0.915 [0.708—1.183]
Pre-treatment confidence	0.049	0.071	0.475	1	.490	1.050 [0.914—1.205]
Pre-treatment readiness	0.070	0.087	0.646	1	.421	1.072 [0.905—1.271]

*Cov* Covariate, *ATSPPH-SF* Attitudes Toward Seeking Professional Psychological Help- Short Form. Gender coded as 0 = men, 1 = women; and race coded as 0 = white, 1 = non-white

Substantive significant predictors are bolded where *denotes significance at 0.05 level; **denotes significance at 0.01 level. The Nagelkerke pseudo *R*^2^ for this model was 0.132

### Predictors of percentage of program completed

Multiple regression analyses were conducted to evaluate the extent to which the conceptual predictor groupings could predict percentage of the program completed. Gender and race were included as covariates in block one for each model. Individual cannabis-specific factors explained approximately 18.4% of the variance in percentage of program completion. Among the factors, greater social motives for cannabis use significantly predicted a greater percentage of the program being completed (see Table 5). Mental health and other substance use factors explained approximately 11% of the variance in percentage of program completion. After controlling for race and gender, no factors significantly predicted a greater percentage of the program being completed (see Table 6). Treatment belief factors explained approximately 14% of the variance in percentage of program completion. More positive attitudes towards treatment seeking significantly predicted a greater percentage of the program being completed (see Table 7).

**Table 5:** Hierarchical regression results for individual cannabis factors predicting percentage of completion

Factor	*B*	95% CI *B*	Std. Error	*t*	*Sig*
Gender (Cov)	5.747	−10.169 – 54. 080	8.093	0.710	.480
Race (Cov)	8.016	−10.370 – 21.865	8.808	0.910	.366
Cannabis problems (CUDIT-R)	0.911	−0.760 – 2.581	0.839	1.086	.281
Family history density	−22.274	−52.880 – 8.333	15.367	−1.449	.151
MMQ Enhancement motives	−9.962	−15.265 – 13.341	7.182	−0.134	.894
MMQ Conformity motives	18.653	−13.446 – 50.751	16.116	1.157	.251
MMQ Expansion motives	−6.719	−17.335 – 3.897	5.330	−1.261	.211
MMQ Coping motives	−3.096	−13.769 – 7.576	5.359	−0.578	.565
MMQ Social motives	**15.815**	**3.361–28.268**	**6.253**	**2.529**	.014*

*Cov* Covariate, *CUDIT-R* Cannabis Use Disorder Identification Test – Revised, *MMQ* Marijuana Motives Questionnaire. Gender coded as 0 = men, 1 = women; and race coded as 0 = white, 1 = non-white

Substantive significant predictors are bolded where *denotes significance at 0.05 level; **denotes significance at 0.01 level. The *R*^*2*^ for the complete model was 0.184

**Table 6:** Hierarchical regression results for mental health and other substance use factors predicting percentage of completion

Factor	*B*	95% CI *B*	Std. Error	*t*	*Sig*
Gender (cov)	5.339	−13.367 – 24.045	9.348	0.571	.570
Race (cov)	23.422	1.463–45.382	10.974	2.134	.037*
Alcohol use frequency (NIAAA)	1.997	−3.721 – 7.715	2.857	0.699	.487
Alcohol use quantity (NIAAA)	−1.502	−9.314 – 6.309	3.904	−0.385	.702
Diagnosed mental illness	−13.846	−39.848 – 12.156	12.995	−1.066	.291
Lifetime mental illness treatment	9.892	−16.940 – 36.724	13.409	0.738	.464
Depression (PHQ-9)	−0.879	−2.392 – 0.633	0.756	−1.163	.249

*Cov* Covariate, *PHQ-9* Patient Health Questionnaire – 9, *NIAAA* National Institute on Alcohol Abuse and Alcoholism’s recommended alcohol use disorder screening questions. Gender coded as 0 = men, 1 = women; and race coded as 0 = white, 1 = non-white

Substantive significant predictors are bolded where *denotes significance at 0.05 level. The *R*^*2*^ for the complete model was 0.110

**Table 7:** Hierarchical regression results for treatment belief factors predicting percentage of completion

Factor	*B*	95% CI *B*	Std. Error	*t*	*Sig*
Gender (Cov)	8.670	−7.156 – 24.497	7.953	1.090	.279
Race (Cov)	9.029	−8.056 – 26.114	8.585	1.052	.296
Attitudes towards treatment (ATSPPH-SF)	**1.738**	**0.468–3.009**	**0.638**	**2.723**	.008*
Pre-treatment importance	−4.312	−10.643 – 2.019	3.181	−1.356	.179
Pre-treatment confidence	−1.683	−5.139 – 1.773	1.737	−0.969	.336
Pre-treatment readiness	2.759	−1.305 – 6.822	2.042	1.351	.181

*Cov* Covariate, *ATSPPH-SF* Attitudes Toward Seeking Professional Psychological Help- Short Form. Gender coded as 0 = men, 1 = women; and race coded as 0 = white, 1 = non-white

Substantive significant predictors are bolded where *denotes significance at 0.05 level; **denotes significance at 0.01 level. The *R*^*2*^ for the complete model was 0.140

## Discussion

One primary observation of this study is that recruiting individuals with heavy cannabis use for treatment is a demonstrated difficult endeavor, particularly in the context of a clinical trial to examine treatment efficacy. While difficulties with recruitment and retention are well-known among researchers, there is minimal published literature that focuses on discussing this phenomenon. The present study offered evidence for some of the difficulties in conducting clinical trials for online treatments, as well as providing detailed information about key participant disengagement points. Only a small proportion (approximately 10%) of those initially in contact with the *CANreduce* program engaged in one of the two *CANreduce* guided introductions and subsequent treatment program. Most of the attrition in the current study took place during eligibility screening. While part of the initial recruitment difficulty may have been drawing on university participant pool subjects where participants may have completed questionnaires for course credit rather than treatment seeking for cannabis use problems (as illustrated by the *n* = 347 for insufficient cannabis use that were excluded from participating), a significant proportion (50%) of individuals who were eligible for the program and hence, had at least moderate cannabis use problems, still did not engage in the offered treatment. Additionally, it is possible that individuals with less than moderate cannabis use were interested in engaging in the program but were screened out due to RCT inclusion and exclusion criteria. Given than a wide spectrum of cannabis use severity can benefit from online, self-guided treatments, future studies could examine the outcomes of offering the individuals with less than moderate severity cannabis use the online modules as a brief intervention without the guided introduction. Beyond the scope of this paper, but important when looking at engagement in the *CANreduce* program, results from the initial *CANreduce* study (Rysen et al., under review) demonstrated 66% participant drop out from study engagement to 10-week follow up using an Intent-to-Treat analysis (inclusion in the ITT population was determined based on contact with facilitators at least once via email). Further, for those individuals who engaged in either the MET-therapist or non-MET research assistant, the average percent of the online *CANreduce* program content completed was 51.58% (*SD* = 36.82) with 24.5% (*n* = 23) of participants completing all 8 modules with no significant differences between the two conditions (Rysen et al., under review). Together, attrition at any stage during the treatment significantly affects participants’ treatment outcomes, as well as the researcher’s ability to appropriately examine the efficacy of treatment programs.

A primary aim of the present paper was to address individual factors that affect treatment initiation and engagement in the *CANreduce* program. Information about the individual factors that affect treatment initiation and engagement has been vastly understudied in treatment programs, particularly in substance use research. By having participants complete comprehensive pre-intervention assessment batteries prior to engaging in the *CANreduce* program, we were able to examine meaningful differences between treatment seekers who showed interest in the program but did not participate, and those who followed through with participation, as well as factors that contributed to engagement in the program, as defined by percentage of the online program completed.

Treatment initiation factors from the present study included higher cannabis use problems score, lower family history density, increased alcohol use frequency, and more positive attitudes towards treatment. Higher cannabis use problems predicting treatment initiation for the program is consistent with literature that suggests individuals who are concerned about the harm caused to them by cannabis use are more likely to seek treatment. (Williamson et al. 2022). Additionally, lower family history density predicted more treatment initiation in the program. Leaning on literature regarding injunctive norms, if cannabis use in an individual’s immediate social circle is normative, the individual may be more likely to accept regular cannabis use as acceptable, or encounter increased barriers to change (e.g., more readily available cannabis, increased motives for cannabis use). Literature suggests that family history density plays an important role in cannabis use behaviours (Khoddam et al., 2015), and that parental CUD (but not parental cannabis use that is not at the CUD level of severity) is associated with adolescent cannabis use (Hill et al. 2018). Alcohol use frequency was found to be a statistically significant treatment initiation factor, where increased alcohol frequency increased the odds of participating in the program. Co-use of alcohol and cannabis can have significantly greater negative outcomes than using either substance on their own (Yurasek et al. 2017), demonstrate heavier use (Thompson et al. 2021) and may increase harms such as drunk driving, social consequences and harm to self (Subbaraman & Kerr 2015). Given the increased severity of symptoms and impact on co-using participants’ lives, they may have been more likely to see treatment as a worthwhile option. Finally, attitudes towards treatment were found to be statistically significant in predicting odds of engaging in the program. Previous literature suggests that cannabis help seekers hold a more positive attitude towards treatments (van der Pol et al. 2013). Given that individuals who have greater treatment expectancies tend to have better treatment outcomes (Raylu & Kaur 2012), especially when paired with client self-efficacy (Kuusisto et al. 2011), it is unsurprising that those with more positive attitudes towards treatment predicted treatment initiation.

Factors predicting engagement in the program, as defined by percentage of the online *CANreduce* program completed, included increased social motives for cannabis use and more positive attitudes towards treatment. Increased social motives predicting increased percentage of the program completed is interesting, given that in other addiction literature, social motives have been shown to hinder treatment seeking efforts as resolving the addiction may reduce the social aspect of the use (Sztainert et al. 2014). However, given that a large proportion of the *CANreduce* program that aimed to address changing normative perceptions of cannabis use among the general population and specifically navigating social situations without cannabis use, perhaps these individuals were able to better engage in the program and were interested in adapting how they were meeting their social needs with cannabis use. It is possible that addressing social motives for cannabis use over the course of the program made the program content more approachable, whereas content addressing other more challenging motives tied more directly to CUD severity (e.g., coping motives, Moitra et al. 2015), may have led to more disengagement in the program. Similar to predicting treatment initiation, positive attitudes towards treatment also predicted increased percentage of program completed. This is consistent with wider literature that suggests individuals who have negative perceptions of care show less engagement in treatment (McLean et al. 2022; Sturgess et al., 2016). Together, the information gleaned about individual treatment initiation and engagement factors adds to a relatively sparse literature regarding substance use treatment programs, and can be used to better implement programs among treatment seekers.

In sum, the present paper illustrates the difficulties in both recruiting and engaging individuals with heavy cannabis use in an online treatment with facilitator contact prior to self-help therapy modules for cannabis use. While there is great need and evidence of effective evidence-based treatment, several barriers to getting an individual to attend treatment exist, as illustrated by the present study’s findings of significant pre-treatment factors affecting treatment initiation and engagement. To combat the difficulties in recruiting and engaging individuals to begin treatment, the authors make the following suggestions. While it is important to control for external factors when examining the efficacy of newly developed treatment programs through inclusion and exclusion criteria characteristic of RCT’s (e.g., including participants with moderate difficulties in target substance areas, and controlling for comorbidities or outside treatment), strict criteria can impact enrollment in the program and narrow the scope on who can potentially benefit from the treatment. Restricting who meets enrollment criteria in the program affects both the researcher’s ability to evaluate the program with a large enough sample size in a timely manner (and make good use of study resources) as well as the potential benefits the participants could gain from completing the program. Several individuals who completed the eligibility survey in the current study had just below moderate cannabis difficulties, and hence were ineligible to participate in the study. However, given the wider literature and the stepped-care approach to treatment, individuals across the substance use intensity spectrum (i.e., mild or moderate towards more severe problems) do not have differential dropout rates from treatment programs (Dacosta-Sanchez et al., 2019) and may benefit from online (self- or guided) treatment (Riper et al. 2014; Eék et al. 2023). Moving towards including a wider range of cannabis use severity may help expand the benefits of cost-effective, easily accessible online treatment programs to a wider range of individuals. Given that online programs can benefit a wide spectrum of cannabis use severities and increased cannabis use problems predicted engagement in the guided introduction and program, future studies could examine the outcomes of admitting individuals with less than moderate cannabis problems directly into the self-guided program, while having individuals with moderate severity or higher cannabis use into a therapist-guided introduction and subsequent program.

Second, while we could not control individual factors affecting participation, more can be done to address factors associated with pre-treatment drop out earlier in the treatment-seeking pathway. As an example, it is only in the second last module of the Canadian *CANreduce* program where navigating cannabis among close social relationships is addressed. Given that family history density (and likely resulting social norms and situations) impacted treatment initiation, providing brief information upon registration (e.g., having a welcome email with topics to expect, or addressing frequently asked questions [i.e., “If my family and friends still use cannabis, is it harder for me to stop?”]) may offset these drop out factors. This may be particularly helpful, given that individuals who initiated the program and had higher social motives for use ended up more fully engaging in the program as illustrated by increased percentage of program completed. Future research should consider tailoring the delivery of intervention content in a method that is more individualized and address individual risk factors earlier in the pathway to mitigate treatment initiation and engagement difficulties (e.g., if coping is the primary reason for cannabis use, providing earlier information on alternative coping methods may be warranted). Approaches to individualized treatment, such as the ecological momentary assessment and intervention (EMA/EMI) has been used in other areas of substance use treatment (e.g., alcohol use), show that tailoring programs to risk factors and delivering that information in a timely manner helps to decrease substance use and associated motives for substance use over time (Blevins et al. 2021). More research is needed to better understand how to intervene earlier in the treatment seeking pathway to address the wide array of pre-treatment drop out factors demonstrated in this study, as well as other studies in various substance use areas. Additionally, more research is needed to examine the differential effects of personal factors on initiation and engagement between in-person and online modalities of treatment. While offering online treatment may overcome some typical barriers to treatment such as stigma (Gates et al., 2016), cost of treatment (Ellingstad et al. 2006), and complicated access in rural or remote areas (Richards & Viganó 2013), some researchers have found differential dropout rates between in person and online treatment formats (Ngo et al. 2022; Sistad et al. 2023). More research is needed to determine if method of delivery for the CANreduce program produces differential effects.

The present study is not without limitations. First, the present study only examined individual-level predictors and did not take into consideration broader sociocultural factors. Important shifts in the sociocultural landscape, such as the post-legalization context in Canada, may have significantly impacted individuals’ perceptions of treatment need, stigma surrounding help-seeking, and access to care (Mennis et al. 2023). Emerging international evidence further underscores this point (Matheson & Le Foll 2020). For example, research examining contemporary cannabis markets has highlighted how increasing potency, greater availability of high-THC products, and more frequent patterns of daily use may influence how individuals perceive the risks associated with cannabis and whether they recognize a need for treatment. A large multicenter case–control study across Europe demonstrated that daily use of high-potency cannabis was associated with increased risk of psychotic disorders at the population level (Di Forti et al. 2019), underscoring the potential clinical implications of contemporary intensive cannabis use patterns. Building on this work, recent analyses from the first NHS clinic specifically developed for young adults with comorbid cannabis use and psychotic disorders have further emphasized the complexity of engaging individuals in treatment in contexts where frequent and high-potency cannabis use has become increasingly normalized (Di Forti et al., 2024). Although the present study did not examine psychosis or related clinical outcomes, this broader body of work provides an important sociocultural framework for understanding how evolving cannabis potency, patterns of use, and perceptions of harm may shape recognition of problematic use and willingness to initiate or engage in treatment. Future research examining engagement in online cannabis interventions such as CANreduce may benefit from integrating these broader contextual factors. For example, studies could assess the THC potency of cannabis products used by participants to better characterize contemporary use patterns, as well as examine psychotic-like experiences associated with cannabis use that may influence individuals’ perceptions of harm or act as barriers to treatment engagement. Given that much of the literature referenced in the present manuscript reflects the pre-legalization era, future studies should more explicitly integrate these broader international trends to better understand engagement and attrition in online cannabis interventions within the post-legalization context. Second, the sample in the present study was comprised of mainly white, young-to-middle aged women who were employed full-time. Given the significant differences in treatment access, cannabis use patterns, and treatment outcomes among populations underrepresented in this study, the generalizability of these results is limited. Future research should aim to more adequately represent a broader segment of the population than that in the present sample. Third, the present study only examined treatment engagement by calculating percentage of program modules completed. While this metric provided a standardized comparison across participants who were given access to the program, several additional indicators could provide a more comprehensive evaluation of engagement, such as use of the cannabis use diary tool, frequency of visits to the website, or self-reported engagement with program materials. A fourth limitation was that the present study did not distinguish between recreational and medical cannabis use purposes. While some measures captured problematic use regardless of intended purpose (e.g., CUDIT-R), other measures could have more precisely assessed reasons for cannabis use (e.g., MMQ). Future research should better delineate recreational and medical cannabis use purposes and further explore how intent of use may affect treatment engagement and dropout risk. Fifth, the present study divided predictors into three conceptual groups (i.e., individual cannabis-specific factors, mental health and other substance use factors, and treatment belief factors). While a comprehensive singular model may help clarify the unique contributions of each predictor, the present study opted for three separate analyses to better balance conceptual clarity and statistical power. Future studies should aim to increase sample size to allow these predictors to be examined simultaneously within a single model.

Overall, our findings reflect the well-documented difficulties in recruiting and engaging participants for online cannabis use research. The present study elucidated several pre-treatment factors that played significant roles in both treatment initiation and treatment engagement in the *CANreduce* treatment program, one of the first online, CBT and MET self-guided treatment programs for heavy cannabis use available in North America that has an integrated MET-therapist guided introduction. Future research should consider using findings from the present study on pre-treatment factors to minimize drop out in the critical period between screening and treatment initiation, as well as to maximize treatment participation and engagement.

## Data Availability

The participants of this study did not give written consent for their data to be shared publicly, so due to the sensitive nature of the research supporting data is not available.

## Abbreviations

ATSPPH-SF: Attitudes Towards Seeking Professional Psychological Help-Short Form
CBT: Cognitive behavioural therapy
CUD: Cannabis Use Disorder
CUDIT-R: Cannabis Use Disorders Identification Test-Revised
ITT: Intent-to-treat
MET: Motivational Enhancement Therapy
MI: Motivational Interviewing
MMQ: Marijuana Motives Questionnaire
NIAAA: National Institute on Alcohol Abuse and Alcoholism
PHQ-9: Patient Health Questionnaire
RCT: Randomized Controlled Trial

## Footnotes

1 Due to the low representation of transgender and non-binary individuals, we coded their data for this variable as missing.

2 Due to variable coding, Table 2 states higher family history density predicts lower treatment initiation. To keep discussion consistent with factors that predict higher treatment initiation, this is discussed in text as lower family history density predicting higher treatment initiation.
